# *N*-(2,3-Dimethyl­phen­yl)benzamide

**DOI:** 10.1107/S1600536809012112

**Published:** 2009-04-08

**Authors:** B. Thimme Gowda, Miroslav Tokarčík, Jozef Kožíšek, B. P. Sowmya, Hartmut Fuess

**Affiliations:** a Department of Chemistry, Mangalore University, Mangalagangotri 574 199, Mangalore, India; b Faculty of Chemical and Food Technology, Slovak Technical University, Radlinského 9, SK-812 37 Bratislava, Slovak Republic; c Institute of Materials Science, Darmstadt University of Technology, Petersenstrasse 23, D-64287 Darmstadt, Germany

## Abstract

The conformation of the N—H bond in the structure of the title compound, C_15_H_15_NO, is *anti* to the *ortho* and *meta*-methyl substituents in the aniline benzene ring, in contrast to the *syn* conformation observed with respect to the *ortho* and *meta*-chloro substituents in *N*-(2,3-dichloro­phen­yl)benzamide. Furthermore, the conformations of N—H and C=O bonds in the amide group are *anti* to each other, similar to those observed in other benzanilides. The dihedral angle between the benzoyl and aniline rings is 84.1 (2)°. The amide group is twisted by 23.0 (3)° out of the plane of the benzoyl ring. The structure exhibits positional disorder over the aniline ring, with site occupancies of 0.80 (1) and 0.20 (1) for the major and minor components, respectively. In the crystal, mol­ecules are connected through N—H⋯O hydrogen bonds into chains running along the *b* axis. An intra­molecular C—H⋯O close contact occurs.

## Related literature

For related structures, see Azumaya *et al.* (1994 ▶); Gowda *et al.* (2003 ▶, 2007 ▶, 2008*a* ▶,*b* ▶).
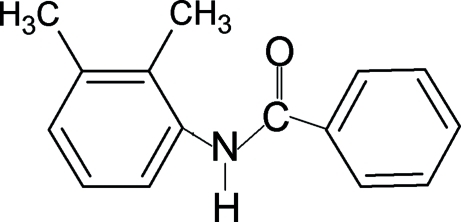


## Experimental

### 

#### Crystal data

C_15_H_15_NO
*M* *_r_* = 225.28
Orthorhombic, 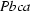

*a* = 8.4656 (2) Å
*b* = 9.4848 (2) Å
*c* = 31.0957 (9) Å
*V* = 2496.81 (11) Å^3^
*Z* = 8
Mo *K*α radiation
μ = 0.08 mm^−1^
*T* = 295 K
0.52 × 0.16 × 0.05 mm

#### Data collection

Oxford Diffraction Xcalibur System diffractometer
Absorption correction: multi-scan (*CrysAlis RED*; Oxford Diffraction, 2008 ▶) *T*_min_ = 0.963, *T*_max_ = 0.996
48994 measured reflections
2409 independent reflections
1572 reflections with *I* > 2σ(*I*)
*R*_int_ = 0.052

#### Refinement

*R*[*F* ^2^ > 2σ(*F* ^2^)] = 0.052
*wR*(*F* ^2^) = 0.146
*S* = 1.05
2409 reflections
222 parameters
14 restraints
H atoms treated by a mixture of independent and constrained refinement
Δρ_max_ = 0.10 e Å^−3^
Δρ_min_ = −0.14 e Å^−3^

### 

Data collection: *CrysAlis CCD* (Oxford Diffraction, 2008 ▶); cell refinement: *CrysAlis RED* (Oxford Diffraction, 2008 ▶); data reduction: *CrysAlis RED*; program(s) used to solve structure: *SHELXS97* (Sheldrick, 2008 ▶); program(s) used to refine structure: *SHELXL97* (Sheldrick, 2008 ▶); molecular graphics: *ORTEP-3* (Farrugia, 1997 ▶) and *DIAMOND* (Brandenburg, 2002 ▶); software used to prepare material for publication: *SHELXL97*, *PLATON* (Spek, 2009 ▶) and *WinGX* (Farrugia, 1999 ▶).

## Supplementary Material

Crystal structure: contains datablocks I, global. DOI: 10.1107/S1600536809012112/dn2440sup1.cif

Structure factors: contains datablocks I. DOI: 10.1107/S1600536809012112/dn2440Isup2.hkl

Additional supplementary materials:  crystallographic information; 3D view; checkCIF report

## Figures and Tables

**Table 1:** Hydrogen-bond geometry (Å, °)

*D*—H⋯*A*	*D*—H	H⋯*A*	*D*⋯*A*	*D*—H⋯*A*
N1—H1*N*⋯O1^i^	0.883 (16)	2.028 (17)	2.907 (2)	173 (2)
C14—H14*C*⋯N1	0.96	2.39	2.852 (4)	109

Symmetry code: (i) 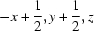.

**Fig. 1.**
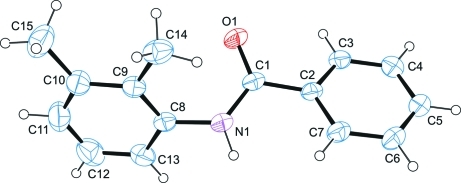
Molecular structure of (I) showing the atom labelling scheme. Displacement ellipsoids are drawn at the 30% probability level. H atoms are represented as small spheres of arbitrary radii. For the sake of clarity, only the major component is represented.

**Fig. 2.**
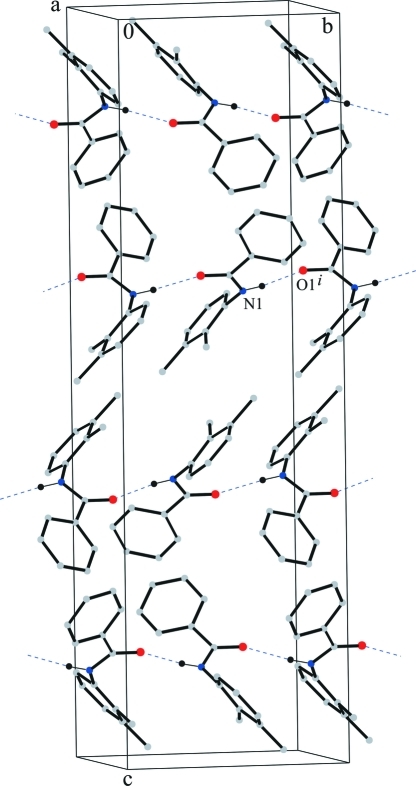
Part of the crystal structure of (I). Molecular chains running along the b axis are generated by N—H···O(i) hydrogen bonds (shown as dashed lines). Symmetry code (i): -x + 1/2,y + 1/2,z. H atoms not involved in intermolecular bonding have been omitted.

**Fig. 3.**
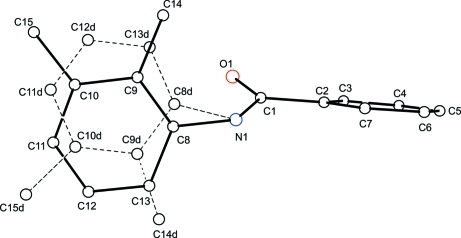
The disorder of the anilino ring in (I). The major component (C8 to C15) has site occupancy factor 0.80. The minor component atoms (C8d to C15d) with site occupancy factor 0.20 has their bonds shown as dashed lines.

## supplementary crystallographic information

### Comment

As part of our continuing efforts to explore the effect of substituents on the solid state geometries of benzanilides (Gowda *et al.*, 2003, 2007, 2008*a*,*b*), in the present work, the structure of *N*-(2,3-dimethylphenyl)benzamide (N23DMPBA) has been determined. The conformation of the N—H bond in N23DMPBA is *anti* to both the *ortho* and *meta*-methyl substituents in the aniline benzene ring (Fig. 1), in contrast to the *syn* conformations observed with respect to both the *ortho* and *meta*-chloro substituents in *N*-(2,3-dichlorophenyl)benzamide (N23DCPBA)(Gowda *et al.*, 2007), while the conformations of the N—H and C=O bonds in the amide group of N23DMPBA are anti to each other, similar to that observed in N23DCPBA, *N*-(2,6-dimethylphenyl)benzamide (Azumaya *et al.*, 1994; Gowda *et al.*, 2008*b*), *N*-(3,4-dimethylphenyl)benzamide (Gowda *et al.*, 2008*a*) and other benzanilides (Gowda *et al.*, 2003). In the crystal structure, the N—H···O hydrogen bonds link the molecules into chains running along the *b* axis (Table 1 & Fig. 2), while the structure is stabilized by C—H···N intramolecular hydrogen bond with atom C14 as donor and atom N1 as acceptor. The atoms of anilino ring (including the methyl groups) are positionally disordered (Fig. 3) with site occupancy factor 0.80 for major component (atoms C8 to C15) and 0.20 for minor component (C8d to C15d). The disordered orientations of anilino ring are essentially planar, forming a dihedral angle of 1.5 (7)°. The dihedral angle between the benzoyl and anilino ring (major) is 84.1 (2)°. The amido group is twisted by 23.0 (3)° out of the plane of benzoyl ring.

### Experimental

The title compound was prepared according to the literature method (Gowda *et al.*, 2003). The purity of the compound was checked by determining its melting point. It was characterized by recording its infrared and NMR spectra. Single crystals of the title compound were obtained from an ethanol solution and used for X-ray diffraction studies at room temperature.

### Refinement

During the refinement of (I) the atoms of anilino ring revealed unusual anisotropic displacement parameters, so we introduced two sets of split sites for this part of molecule. The positions of methyl groups were carefully localized (Fig.3). Final cycles of refinement were done with fixed site occupancy factors (0.80 and 1/5) and the following restraints: The major component of the disorder (atoms C8 to C15) was refined free, except for DELU restraint imposed on the atoms C8, C10. The minor component was subject to restraint on the geometry of the ring (rigid planar hexagon) and restraint on the anisotropic displacement parameters - using DELU instruction for ring atoms. As to the hydrogen atoms, the amido H atom was seen in difference maps and its positional parameters were refined with the restraint on N—H distance, set at 0.86 (2) Å. All other H atoms were placed in geometrically idealized positions and constrained to ride on their parent atoms with C—H distances in the range 0.93–0.96 Å and with displacement parameters *U*_iso_(H) set at 1.2 *U*_eq_(C-aromatic,*N*) or 1.5 *U*_eq_(*C*-methyl).

### Crystal data

**Table:**

C_15_H_15_NO	*F*(000) = 960
*M**_r_* = 225.28	*D*_x_ = 1.199 Mg m^−^^3^
Orthorhombic, *P**b**c**a*	Mo *K*α radiation, λ = 0.71073 Å
Hall symbol: -P 2ac 2ab	Cell parameters from 12625 reflections
*a* = 8.4656 (2) Å	θ = 3.1–29.2°
*b* = 9.4848 (2) Å	µ = 0.08 mm^−^^1^
*c* = 31.0957 (9) Å	*T* = 295 K
*V* = 2496.81 (11) Å^3^	Rod, colourless
*Z* = 8	0.52 × 0.16 × 0.05 mm

### Data collection

**Table:**

Oxford Diffraction Xcalibur System diffractometer	2409 independent reflections
graphite	1572 reflections with *I* > 2σ(*I*)
Detector resolution: 10.434 pixels mm^-1^	*R*_int_ = 0.052
ω scans with κ offsets	θ_max_ = 25.9°, θ_min_ = 3.3°
Absorption correction: multi-scan (*CrysAlis RED*; Oxford Diffraction, 2008)	*h* = −10→10
*T*_min_ = 0.963, *T*_max_ = 0.996	*k* = −11→11
48994 measured reflections	*l* = −38→38

### Refinement

**Table:**

Refinement on *F*^2^	Primary atom site location: structure-invariant direct methods
Least-squares matrix: full	Secondary atom site location: difference Fourier map
*R*[*F*^2^ > 2σ(*F*^2^)] = 0.052	Hydrogen site location: inferred from neighbouring sites
*wR*(*F*^2^) = 0.146	H atoms treated by a mixture of independent and constrained refinement
*S* = 1.05	*w* = 1/[σ^2^(*F*_o_^2^) + (0.0655*P*)^2^ + 0.6303*P*] where *P* = (*F*_o_^2^ + 2*F*_c_^2^)/3
2409 reflections	(Δ/σ)_max_ < 0.001
222 parameters	Δρ_max_ = 0.10 e Å^−^^3^
14 restraints	Δρ_min_ = −0.14 e Å^−^^3^

### Special details

**Table:**

Geometry. All e.s.d.'s (except the e.s.d. in the dihedral angle between two l.s. planes) are estimated using the full covariance matrix. The cell e.s.d.'s are taken into account individually in the estimation of e.s.d.'s in distances, angles and torsion angles; correlations between e.s.d.'s in cell parameters are only used when they are defined by crystal symmetry. An approximate (isotropic) treatment of cell e.s.d.'s is used for estimating e.s.d.'s involving l.s. planes.
Refinement. Refinement of *F*^2^ against ALL reflections. The weighted *R*-factor *wR* and goodness of fit *S* are based on *F*^2^, conventional *R*-factors *R* are based on *F*, with *F* set to zero for negative *F*^2^. The threshold expression of *F*^2^ > σ(*F*^2^) is used only for calculating *R*-factors(gt) *etc*. and is not relevant to the choice of reflections for refinement. *R*-factors based on *F*^2^ are statistically about twice as large as those based on *F*, and *R*- factors based on ALL data will be even larger.

### Fractional atomic coordinates and isotropic or equivalent isotropic displacement parameters (Å^2^)

**Table:**

	*x*	*y*	*z*	*U*_iso_*/*U*_eq_	Occ. (<1)
N1	0.2585 (2)	0.60702 (17)	0.37113 (6)	0.0562 (5)	
H1N	0.273 (3)	0.6973 (18)	0.3654 (7)	0.067*	
O1	0.1702 (2)	0.39655 (14)	0.34744 (5)	0.0676 (5)	
C1	0.1605 (2)	0.52512 (19)	0.34747 (6)	0.0496 (5)	
C2	0.0397 (2)	0.59716 (19)	0.32076 (6)	0.0461 (5)	
C3	−0.0200 (2)	0.5254 (2)	0.28599 (7)	0.0549 (5)	
H3	0.0147	0.4342	0.2804	0.066*	
C4	−0.1302 (3)	0.5859 (2)	0.25935 (7)	0.0635 (6)	
H4	−0.1678	0.5369	0.2356	0.076*	
C5	−0.1844 (3)	0.7196 (2)	0.26811 (8)	0.0701 (7)	
H5	−0.2597	0.7609	0.2504	0.084*	
C6	−0.1277 (3)	0.7913 (2)	0.30275 (8)	0.0729 (7)	
H6	−0.1657	0.8812	0.3087	0.087*	
C7	−0.0153 (3)	0.7326 (2)	0.32897 (7)	0.0581 (6)	
H7	0.0242	0.7833	0.3522	0.07*	
C8	0.3938 (5)	0.5547 (4)	0.39290 (14)	0.0517 (10)	0.8
C9	0.3754 (4)	0.4635 (4)	0.42634 (13)	0.0567 (8)	0.8
C10	0.5108 (6)	0.4031 (5)	0.44545 (16)	0.0718 (10)	0.8
C11	0.6555 (6)	0.4480 (7)	0.4303 (2)	0.0842 (13)	0.8
H11	0.7458	0.4096	0.4427	0.101*	0.8
C12	0.6745 (5)	0.5444 (5)	0.39867 (14)	0.0910 (11)	0.8
H12	0.7754	0.5718	0.3903	0.109*	0.8
C13	0.5457 (5)	0.6012 (5)	0.37902 (16)	0.0713 (12)	0.8
H13	0.5563	0.668	0.3573	0.086*	0.8
C14	0.2156 (5)	0.4279 (4)	0.44389 (12)	0.0748 (10)	0.8
H14A	0.2122	0.4502	0.474	0.112*	0.8
H14B	0.1955	0.3292	0.4399	0.112*	0.8
H14C	0.1367	0.4817	0.429	0.112*	0.8
C15	0.4970 (6)	0.2939 (4)	0.48009 (12)	0.1080 (14)	0.8
H15A	0.4326	0.2174	0.4701	0.162*	0.8
H15B	0.4493	0.3352	0.5051	0.162*	0.8
H15C	0.6002	0.2593	0.4873	0.162*	0.8
C8D	0.3513 (13)	0.5282 (12)	0.4010 (3)	0.051 (4)	0.2
C9D	0.5124 (14)	0.5487 (12)	0.3951 (3)	0.053 (3)	0.2
C10D	0.6201 (10)	0.4779 (17)	0.4211 (5)	0.082 (5)	0.2
C11D	0.5667 (12)	0.3865 (16)	0.4528 (5)	0.078 (5)	0.2
H11D	0.6387	0.3392	0.4702	0.094*	0.2
C12D	0.4055 (14)	0.3660 (9)	0.4587 (3)	0.067 (3)	0.2
H12D	0.3698	0.3048	0.4799	0.081*	0.2
C13D	0.2978 (10)	0.4368 (12)	0.4328 (3)	0.048 (3)	0.2
H13D	0.19	0.423	0.4367	0.057*	0.2
C14D	0.5865 (19)	0.648 (2)	0.3617 (7)	0.090 (6)	0.2
H14D	0.5112	0.6671	0.3393	0.135*	0.2
H14E	0.6786	0.6049	0.3496	0.135*	0.2
H14F	0.6157	0.7352	0.3754	0.135*	0.2
C15D	0.7999 (14)	0.4840 (15)	0.4207 (5)	0.081 (4)	0.2
H15D	0.8375	0.4718	0.3918	0.121*	0.2
H15E	0.8414	0.4102	0.4386	0.121*	0.2
H15F	0.8343	0.5737	0.4315	0.121*	0.2

### Atomic displacement parameters (Å^2^)

**Table:**

	*U*^11^	*U*^22^	*U*^33^	*U*^12^	*U*^13^	*U*^23^
N1	0.0656 (11)	0.0354 (8)	0.0678 (11)	−0.0015 (8)	−0.0083 (9)	0.0018 (8)
O1	0.0834 (11)	0.0337 (8)	0.0856 (11)	0.0058 (7)	−0.0171 (9)	−0.0005 (7)
C1	0.0582 (12)	0.0324 (10)	0.0582 (12)	0.0000 (9)	0.0030 (10)	0.0016 (9)
C2	0.0481 (11)	0.0361 (10)	0.0540 (11)	−0.0031 (8)	0.0055 (9)	0.0046 (9)
C3	0.0558 (12)	0.0377 (10)	0.0711 (14)	−0.0031 (9)	−0.0009 (11)	−0.0025 (10)
C4	0.0614 (14)	0.0581 (13)	0.0710 (15)	−0.0079 (11)	−0.0129 (11)	−0.0011 (11)
C5	0.0702 (15)	0.0527 (13)	0.0874 (17)	−0.0020 (11)	−0.0219 (13)	0.0114 (13)
C6	0.0815 (16)	0.0444 (12)	0.0928 (18)	0.0126 (12)	−0.0174 (14)	0.0009 (12)
C7	0.0700 (14)	0.0386 (11)	0.0659 (13)	0.0030 (10)	−0.0054 (11)	−0.0014 (10)
C8	0.053 (3)	0.0387 (15)	0.063 (2)	0.0004 (17)	−0.0032 (18)	−0.0055 (14)
C9	0.067 (2)	0.0446 (18)	0.059 (2)	−0.0009 (18)	−0.005 (2)	−0.0083 (15)
C10	0.076 (3)	0.064 (2)	0.076 (3)	0.003 (2)	−0.015 (2)	−0.0052 (19)
C11	0.081 (3)	0.088 (3)	0.083 (3)	0.015 (3)	−0.023 (3)	−0.006 (2)
C12	0.060 (2)	0.101 (3)	0.112 (3)	−0.012 (2)	0.003 (2)	−0.009 (2)
C13	0.057 (3)	0.070 (3)	0.088 (4)	−0.014 (2)	−0.003 (3)	−0.006 (2)
C14	0.094 (3)	0.066 (2)	0.064 (2)	−0.009 (2)	0.008 (2)	0.0078 (17)
C15	0.150 (4)	0.085 (3)	0.088 (3)	0.019 (3)	−0.035 (2)	0.018 (2)
C8D	0.050 (7)	0.067 (10)	0.037 (7)	−0.007 (7)	−0.002 (6)	0.006 (6)
C9D	0.053 (7)	0.053 (9)	0.052 (8)	−0.020 (6)	0.018 (5)	−0.025 (5)
C10D	0.052 (8)	0.085 (16)	0.108 (17)	−0.008 (7)	−0.015 (7)	−0.031 (9)
C11D	0.061 (7)	0.090 (12)	0.083 (12)	0.029 (9)	−0.026 (9)	−0.020 (7)
C12D	0.082 (8)	0.061 (7)	0.059 (7)	0.005 (6)	−0.019 (6)	0.013 (5)
C13D	0.037 (7)	0.052 (8)	0.054 (9)	−0.001 (7)	0.002 (7)	0.012 (5)
C14D	0.051 (9)	0.083 (12)	0.135 (16)	−0.009 (7)	0.029 (9)	0.033 (10)
C15D	0.046 (7)	0.101 (10)	0.096 (10)	−0.004 (6)	−0.009 (7)	0.022 (8)

### Geometric parameters (Å, °)

**Table:**

N1—C1	1.354 (3)	C12—H12	0.93
N1—C8	1.419 (4)	C13—H13	0.93
N1—C8D	1.428 (4)	C14—H14A	0.96
N1—H1N	0.883 (16)	C14—H14B	0.96
O1—C1	1.222 (2)	C14—H14C	0.96
C1—C2	1.484 (3)	C15—H15A	0.96
C2—C3	1.374 (3)	C15—H15B	0.96
C2—C7	1.390 (3)	C15—H15C	0.96
C3—C4	1.373 (3)	C8D—C9D	1.39
C3—H3	0.93	C8D—C13D	1.39
C4—C5	1.376 (3)	C9D—C10D	1.39
C4—H4	0.93	C9D—C14D	1.538 (16)
C5—C6	1.361 (3)	C10D—C11D	1.39
C5—H5	0.93	C10D—C15D	1.523 (15)
C6—C7	1.372 (3)	C11D—C12D	1.39
C6—H6	0.93	C11D—H11D	0.93
C7—H7	0.93	C12D—C13D	1.39
C8—C9	1.361 (4)	C12D—H12D	0.93
C8—C13	1.426 (6)	C13D—H13D	0.93
C9—C10	1.412 (6)	C14D—H14D	0.96
C9—C14	1.497 (5)	C14D—H14E	0.96
C10—C11	1.379 (7)	C14D—H14F	0.96
C10—C15	1.499 (5)	C15D—H15D	0.96
C11—C12	1.353 (6)	C15D—H15E	0.96
C11—H11	0.93	C15D—H15F	0.96
C12—C13	1.361 (6)		
			
C1—N1—C8	123.6 (2)	C10—C11—H11	117.9
C1—N1—C8D	113.0 (6)	C11—C12—C13	119.9 (4)
C1—N1—H1N	122.1 (14)	C11—C12—H12	120
C8—N1—H1N	108.8 (15)	C13—C12—H12	120
C8D—N1—H1N	124.2 (15)	C12—C13—C8	117.6 (4)
O1—C1—N1	122.11 (19)	C12—C13—H13	121.2
O1—C1—C2	120.35 (18)	C8—C13—H13	121.2
N1—C1—C2	117.54 (16)	C9D—C8D—C13D	120
C3—C2—C7	118.64 (19)	C9D—C8D—N1	112.4 (8)
C3—C2—C1	117.75 (17)	C13D—C8D—N1	127.6 (8)
C7—C2—C1	123.60 (18)	C10D—C9D—C8D	120
C4—C3—C2	121.16 (19)	C10D—C9D—C14D	114.9 (11)
C4—C3—H3	119.4	C8D—C9D—C14D	125.1 (11)
C2—C3—H3	119.4	C9D—C10D—C11D	120
C3—C4—C5	119.5 (2)	C9D—C10D—C15D	129.3 (10)
C3—C4—H4	120.3	C11D—C10D—C15D	110.7 (10)
C5—C4—H4	120.3	C12D—C11D—C10D	120
C6—C5—C4	120.0 (2)	C12D—C11D—H11D	120
C6—C5—H5	120	C10D—C11D—H11D	120
C4—C5—H5	120	C11D—C12D—C13D	120
C5—C6—C7	120.8 (2)	C11D—C12D—H12D	120
C5—C6—H6	119.6	C13D—C12D—H12D	120
C7—C6—H6	119.6	C12D—C13D—C8D	120
C6—C7—C2	119.9 (2)	C12D—C13D—H13D	120
C6—C7—H7	120.1	C8D—C13D—H13D	120
C2—C7—H7	120.1	C9D—C14D—H14D	109.5
C9—C8—N1	119.6 (4)	C9D—C14D—H14E	109.5
C9—C8—C13	122.0 (3)	H14D—C14D—H14E	109.5
N1—C8—C13	118.3 (3)	C9D—C14D—H14F	109.5
C8—C9—C10	119.1 (4)	H14D—C14D—H14F	109.5
C8—C9—C14	121.6 (4)	H14E—C14D—H14F	109.5
C10—C9—C14	119.2 (4)	C10D—C15D—H15D	109.5
C11—C10—C9	116.9 (4)	C10D—C15D—H15E	109.5
C11—C10—C15	121.8 (5)	H15D—C15D—H15E	109.5
C9—C10—C15	121.3 (4)	C10D—C15D—H15F	109.5
C12—C11—C10	124.2 (4)	H15D—C15D—H15F	109.5
C12—C11—H11	117.9	H15E—C15D—H15F	109.5
			
C8—N1—C1—O1	9.3 (4)	C8—C9—C10—C15	−175.3 (4)
C8D—N1—C1—O1	−10.7 (6)	C14—C9—C10—C15	5.9 (7)
C8—N1—C1—C2	−170.1 (3)	C9—C10—C11—C12	−0.4 (8)
C8D—N1—C1—C2	169.9 (6)	C15—C10—C11—C12	178.8 (6)
O1—C1—C2—C3	−22.5 (3)	C10—C11—C12—C13	−1.4 (9)
N1—C1—C2—C3	156.95 (19)	C11—C12—C13—C8	−0.4 (8)
O1—C1—C2—C7	157.7 (2)	C9—C8—C13—C12	4.0 (6)
N1—C1—C2—C7	−22.8 (3)	N1—C8—C13—C12	−176.6 (4)
C7—C2—C3—C4	0.9 (3)	C1—N1—C8D—C9D	122.9 (7)
C1—C2—C3—C4	−178.90 (19)	C8—N1—C8D—C9D	−2.8 (14)
C2—C3—C4—C5	−1.5 (3)	C1—N1—C8D—C13D	−55.9 (9)
C3—C4—C5—C6	0.6 (4)	C8—N1—C8D—C13D	178 (3)
C4—C5—C6—C7	0.8 (4)	C13D—C8D—C9D—C10D	0
C5—C6—C7—C2	−1.3 (4)	N1—C8D—C9D—C10D	−178.9 (12)
C3—C2—C7—C6	0.5 (3)	C13D—C8D—C9D—C14D	−179.0 (17)
C1—C2—C7—C6	−179.7 (2)	N1—C8D—C9D—C14D	2.1 (18)
C1—N1—C8—C9	−66.2 (4)	C8D—C9D—C10D—C11D	0
C8D—N1—C8—C9	−2.2 (18)	C14D—C9D—C10D—C11D	179.1 (15)
C1—N1—C8—C13	114.4 (4)	C8D—C9D—C10D—C15D	−179.8 (18)
C8D—N1—C8—C13	178 (2)	C14D—C9D—C10D—C15D	−0.7 (19)
N1—C8—C9—C10	174.8 (4)	C9D—C10D—C11D—C12D	0
C13—C8—C9—C10	−5.8 (6)	C15D—C10D—C11D—C12D	179.8 (15)
N1—C8—C9—C14	−6.4 (6)	C10D—C11D—C12D—C13D	0
C13—C8—C9—C14	173.0 (4)	C11D—C12D—C13D—C8D	0
C8—C9—C10—C11	3.9 (6)	C9D—C8D—C13D—C12D	0
C14—C9—C10—C11	−174.9 (4)	N1—C8D—C13D—C12D	178.7 (14)

### Hydrogen-bond geometry (Å, °)

**Table:**

*D*—H···*A*	*D*—H	H···*A*	*D*···*A*	*D*—H···*A*
N1—H1N···O1^i^	0.88 (2)	2.03 (2)	2.907 (2)	173 (2)
C14—H14C···N1	0.96	2.39	2.852 (4)	109

Symmetry codes: (i) −*x*+1/2, *y*+1/2, *z*.
